# Poly (α-Dodecyl γ-Glutamate) (PAAG-12) and Polylactic Acid Films Charged with α-Tocopherol and Their Antioxidant Capacity in Food Models

**DOI:** 10.3390/antiox8080284

**Published:** 2019-08-06

**Authors:** Juliana Villasante, Eva Codina, Gádor Indra Hidalgo, Antxon Martínez de Ilarduya, Sebastián Muñoz-Guerra, María Pilar Almajano

**Affiliations:** Chemical Engineering Department, Universitat Politècnica de Catalunya, Av.Diagonal 647, 08028 Barcelona, Spain

**Keywords:** active film, food packaging, characterization, NMR analyses, controlled release, antioxidant, emulsion, food simulant, polymer

## Abstract

Poly (α-dodecyl γ-glutamate) (PAAG-12) was successfully synthesized from poly(γ-glutamic acid) (PGGA) according to Nuclear Magnetic Resonance (NMR) analyses. PAAG-12 films were prepared and enriched with 5% α-tocopherol, with the aim of using them as novel antioxidant active packaging for food applications. Thermogravimetric Analysis (TGA) characterization determined that α-tocopherol improved thermal stability of films, which is beneficial for industrial processing. Polylactic Acid (PLA) films prepared with the same amount of α-tocopherol were used to set a comparative frame and both types of films were applied to two different food models to assess their protective action against oxidation. Water, 50% ethanol (EtOH) and 95% EtOH were used as food simulants and HPLC analyses were performed to determine diffusion and partition coefficients in PLA and the novel polymer, the latter exhibiting slower release rates. Primary oxidation was measured with peroxide value, which revealed that PAAG-12 films with α-tocopherol protected oil-in-water (O/W) emulsions up to 29 days, complying with the Codex Alimentarius.

## 1. Introduction

Oxidation taking place in both raw and processed foods is a current concern to the food industry and market and, therefore, to researchers in this field. When the product is degraded, several oxidation products are created and the quality of food is decreased, due to texture and/or color change, taste depreciation and reduction in nutritional quality. Traditionally, synthetic antioxidants were directly added to food systems to protect them from oxidation. However, years of intense research have come up with a shift in this original idea, and synthetic antioxidants are currently being replaced by natural alternatives for food conservation [1,2].

There is a wide variety of antioxidants that can effectively protect food systems from oxidation. α-tocopherol is a lipophilic natural antioxidant used as an additive in many foods and cosmetics. It is the most active form of vitamin E, which acts as an antioxidant reacting with free radicals solubilized in membrane lipids. Its active center is located at the hydroxyl group in position six of the aromatic ring [3]. 

Ongoing research focuses on new methods to maintain properties of products by using natural antioxidants delivered gradually, which has been demonstrated to improve the efficiency of the antioxidant [4]. This progressive transmission of the additive is achieved by its loading to a matrix or carrier, which is usually a polymer film to simplify the applicability to food packaging, coming up with the term active packaging [5,6,7].

This concept is gaining a lot of popularity specifically when the matrix is a biopolymer, due to several advantages such as the non-toxicity and environmental concern. Among the wide range of available biopolymers, Polylactic Acid (PLA) is a renewable, biodegradable polyester easily obtained from carbohydrate sources such as corn starch, sugar cane and biomass byproducts [8]. It is used for multiple applications and exhibits good mechanical properties even when natural antioxidants are added [9,10,11].

The inclusion of α-tocopherol in PLA films and the subsequent application in food models is a widely studied topic [12,13,14,15], but there are other polymers with promising characteristics that could be interesting carriers for this antioxidant, and that are currently being studied [16]. 

Comblike polymers are a special type of branched polymers that are attracting a lot of attention recently due to their ability to order in a periodical layered structure [17]. Poly(α-dodecyl γ-glutamate) (PAAG-12) is obtained from poly(γ-glutamic acid) (PGGA) by a two-step esterification. The latter is synthesized by bacteria of the genus *Bacillus*, which can be produced as a secretion product, either kept retained in the microorganism capsule or liberated in the medium [18].

In this study, PAAG-12 and PLA films were prepared and charged with 5% (*w*/*w*) α-tocopherol. This amount was the optimum considering that a higher concentration turned the films too brittle for manipulation and lacked applicability. The main goal of this study was to analyze the antioxidant activity against oil-in-water emulsions, both of the new synthesized film (PAGG-12) and of the PLA with α-tocopherol, and to analyze the delivery of α-tocopherol in different situations. Results showed that PAAG-12 has a slower release behavior than PLA and has proved to be a suitable film matrix for α-tocopherol release into oily foods.

To our knowledge, this is the first study where PAAG-12 is applied as an active film for the conservation of food models.

## 2. Materials and Methods

### 2.1. Chemicals

Semicrystalline polylactic acid (PLA), Mw ≈ 140,000 g/mol; Poly(γ-glutamic acid) (PGGA) was supplied by Meiji Co., (Tokyo, Japan). The following reactants were purchased at Sigma-Aldrich Company Ltd.: 1-dodecanol (CH_3_(CH_2_)_11_OH), >98%; α-tocopherol (C_29_H_50_O_2_); ethyl bromide (C_2_H_5_Br), 98%; titanium tetrabutoxide (Ti(OBu)_4_), 97%; iron (II) chloride tetrahydrate (FeCl_2_·4H_2_O), 99%; alumina (aluminum oxide) (Al_2_O_3_), 98%; hydrochloric acid (HCl), 37%; deuterated chloroform (CDCl_3_), 99% *D* atoms; trichloroacetic acid (TCA) (Cl_3_CCOOH), 99%; trifluoroacetic acid (TFA) (CF_3_COOH), 99%; trimethylchlorosilane ((CH_3_)_3_SiCl), >98%; sodium carbonate (Na_2_CO_3_), >99%.

Sodium bicarbonate (NaHCO_3_), 99%; Polyoxyethylene (20) sorbitan monolaurate (Tween-20) (C_58_H_114_O_26_); solvent N-methylpyrrolidone (NMP) (C_5_H_11_N), 97%; and chloroform (CHCl_3_), >99%, were bought at Merck (Darmstadt, Germany). Ammonium thiocyanate (NH_4_SCN), 98%; toluene (C_6_H_5_CH_3_), >98%, were purchased at Panreac Química S.L.U (Barcelona, Spain). Ethanol (CH_3_CH_2_OH), 96%, was purchased at Solvech. Sunflower oil was purchased from a local supermarket.

### 2.2. Preparation of Films

First, 5 g of Poly(γ-glutamic acid) (PGGA) was suspended in 0.2 L of N-methylpyrrolidone and stirred at 80 °C for 2 h. Then the mixture was cooled to 60 °C and 13.15 g (156 mmol) of NaHCO_3_ and 15 mL of ethyl bromide were added (the latter in a continuous flow of 3 mL/h over 5 h). The mixture was stirred at 60 °C for 22 h. The ethylation reaction was followed by ^1^H Nuclear Magnetic Resonance (NMR). After removing the NaBr precipitate by filtration, the reaction solution was poured into 1.5 L of cool HCl (pH = 1.5) to precipitate PAAG-2, which was then separated by filtration. The resulting powder was repeatedly washed with cool water and finally dried under vacuum at 50 °C.

PAAG-2 and 1-dodecanol were mixed at 1:35 molar ratio with Ti(OBu)_4_ in 10% molar of the polymer amount and left to stir under nitrogen atmosphere at 190 °C for 8 h. The reaction progress was monitored by ^1^H-NMR. Once the reaction was finished, the final solution was poured into ethanol at 40 °C. The mixture was further stirred for 2 h and was left to cool down at room temperature. The precipitated polymer (PAAG-12) was separated from the supernatant solution by filtration. This polymer was then dissolved in chloroform to purify it and this step was repeated twice. Purification was accomplished by re-precipitation with methanol and finally dried under vacuum to obtain a white powder.

Once the polymer was obtained, chloroform was used as dissolvent to prepare the film, with a ratio of 12 g of polymer for every 100 mL. Then 5% (*w*/*w*) α-tocopherol was prepared by diluting 500 mg of α-tocopherol in 5 mL of chloroform. It was added to the mixture and then spread onto Petri dishes (5 cm Ø, 0.3 g each) and left to dry at room temperature for 24 h. A film without α-tocopherol was also prepared. 

^1^H and ^13^C NMR spectra were measured on a Nuclear Magnetic Resonance spectrometer (Bruker, model AMX-300, Germany); operating at 300 MHz for ^1^H and 75 MHz for ^13^C. All spectra were obtained in CDCl_3_/TFA, with chemical shifts expressed in ppm and coupling constant (J) in Hertz (Hz).

To prepare the PLA film, semicrystalline PLA (5.125 g) was dissolved in chloroform (100 mL) and stirred until complete dissolution of the polymer. 5% α-tocopherol was added and the homogenized mixture was spread onto Petri dishes (11 cm Ø, 1.025 g each) and left to dry at room temperature. A negative control was prepared (without α-tocopherol).

### 2.3. Characterization of PAAG-12 Films

PAAG-12 films were characterized by Thermogravimetric Analysis using a thermogravimetric analyzer (METTLER-TOLEDO, model DSC/TGA 1 StarSystem), and by Differential Scanning Calorimetry (DSC) using a differential scanning calorimeter (PEKIN ELMER, model PYRIS 1 DSC). The antioxidant they contain, α-tocopherol, was also characterized in order to provide a comparison between films with and without the natural additive. SEM micrographics were also performed to observe the superficial morphology of both PAAG-12 and PLA films. Film thickness was determined.

To perform Thermogravimetric Analysis (TGA), samples of 10–15 mg of α-tocopherol, PAAG-12 film with and without α-tocopherol were introduced in aluminum cups. The samples were heated from 25 to 600 °C with a heating rate of 10 °C/min. The gas flow during the TGA was 20 mL/min, and it was performed in both air (21% O_2_) and nitrogen atmospheres to observe the differences.

DSC analysis thermograms were obtained from 4–6 mg samples at heating and cooling rates of 10 °C·min^−1^ under a nitrogen flow of 20 mL·min^−1^. Indium and zinc were used as standards for temperature and enthalpy calibration. The samples were heated from −60 to 150 °C at a speed of 10 °C/min. The temperature was maintained for 1 min before decreasing it again to the starting point and keeping it there for 1 min. The initial temperature rise was repeated once more, and after 1 min it was decreased to −75 °C at 150 °C/min. This temperature was maintained for 5 min and then raised again at 20 °C/min until 150 °C. Finally, the calorimeter was left to cool at room temperature. For α-tocopherol, the DSC procedure involved a temperature increase from −60 to 150 °C at 10 °C/min and subsequent cooling at room temperature. 

### 2.4. Preparation of Food Simulants

The first food model consisted of three solvents: 2 food simulants (water and 50% aqueous ethanol) and 95% aqueous ethanol Each of them represented different hydrophobicity level, and therefore varying capability of extracting lipophilic substances from the carrier, such as α-tocopherol from PAAG-12 and PLA films. The 2 food simulants were selected according to European Commission regulation (EU) No 10/2011 on plastic materials and articles intended to come into contact with food. The 95% aqueous ethanol was used to simulate extreme conditions such as very fatty foods, high temperatures, etc., because it is a concentration that can also be used.

To perform experiments using these solvents, loaded films were cut in rectangular pieces with a total area of 4 cm^2^. Food simulant was then added to vials and substances left to migrate naturally at a constant temperature (30 °C) and mild shaking speed. The liquid volume/area ratio was 6.25 mL/cm^2^ complying with ASTM D4754-98, which establishes a ratio between 155 and 0.31 mL/cm^2^. Daily samples were taken over 13 days from the vials to analyze with HPLC the amount of α-tocopherol present in the food simulant and were kept frozen (−80 °C). This time span was selected to enable the observation of the slow release with time of the antioxidant from the matrix. 

Samples were injected directly into an HPLC (Waters Alliance 2695 HPLC) with Diode-Array Detector Waters 996, equipped with an autosampler. The column was Ultrabase 100, 3 microns, ODS2 100 × 4.6 mm (Akady). The mobile phase was 100% methanol, therefore isocratic procedure was employed, at a flow rate of 0.8 mL/min. The test lasted 12 min and the injection volume was 20 μL. Signals at a wavelength of 292 nm were stored and collected by Software Empower (Waters). 

#### Estimation of Partition Coefficients and Diffusivities

Results of the release study were quantified with the partition coefficient (*K_P,f_*) and diffusivity (*D*) for each type of film.

From the three general cases of mass transfer theory [19], the one selected was the one in which the film is in contact with an infinite volume of food/simulant and the external mass transfer coefficient is negligible. This assumption is done considering *K_P,f_* < 1, when the migrant, in this research α-tocopherol, has higher affinity towards the food system (this antioxidant is lipophilic therefore has an affinity for targeted oily foods).

The estimation of diffusivities can be performed using Fickian diffusion models, in which simplifications can be made [20] and the equation becomes Equation (1):

(1) $$\frac{M_{f,t}}{M_{f,\infty }}=1-\frac{8}{\pi^{2}}\overset{\infty }{\underset{n=0}{\sum }}\frac{1}{{\left(2n+1\right)}^{2}}\exp\left[-\frac{{\left(2n+1\right)}^{2}}{4L^{2}}D\pi^{2}t\right]$$

where *M_f,t_* is the mass of the migrant in the food (simulant) at a particular time *t*, *M_f,∞_* is the mass of the migrant in the food (simulant) at equilibrium, *L* is the film thickness, *D* is the migrant diffusivity and t is time (International System of Units, SI). This equation can be further simplified when *M_t_*/*M_p_* is <0.6, obtaining Equation (2) [21]:

(2) $$\frac{M_{f,t}}{M_{P}}=\frac{4}{L}{\left(\frac{Dt}{\pi }\right)}^{0.5}$$

where *M_P_* is the initial amount of migrant in the film.

The partition coefficient *K_P,f_* is defined as the ratio of the migrant concentration in the film (*C_p,∞_*) to the migrant concentration in the food simulant (*C_f,∞_*), both at equilibrium:

(3) $$K_{P,f}=\frac{C_{p,\infty }}{C_{f,\infty }}$$

(migration results are presented using D (m^2^/s) and *K_P,f_* values).

### 2.5. Preparation of Oil-In-Water (O/W) Emulsions and Determination of Primary Oxidation

Oil-in-water emulsions were prepared using sunflower oil according to previous research [22] and served as the second food model. Samples were analyzed by peroxide value.

Primary oxidation was assessed by peroxide value using ferric thiocyanate method with calibration using official method of the Association of Official Analytical Chemists (AOAC)’, according to methodology described in previous research [23]. Analyses were performed using UV/Vis spectrum absorbance spectrometer (FLUOstar Omega), equipped with a temperature-controlled incubation chamber and a fluorescence multiplate reader (BMG Labtech, Ortenberg, Germany). Results are expressed as milliequivalents (meq) of hydroperoxide per kg of emulsion.

### 2.6. Statistical Analyses

Peroxide value was determined in triplicate. Data were analyzed using one-way analysis of variance (ANOVA) and Tukey’s comparison test was used to determine significantly different values (*p* < 0.01) using Minitab 18 software.

## 3. Results and Discussion

### 3.1. PAAG-12 Synthesis and Film Preparation

**Figure 1:**
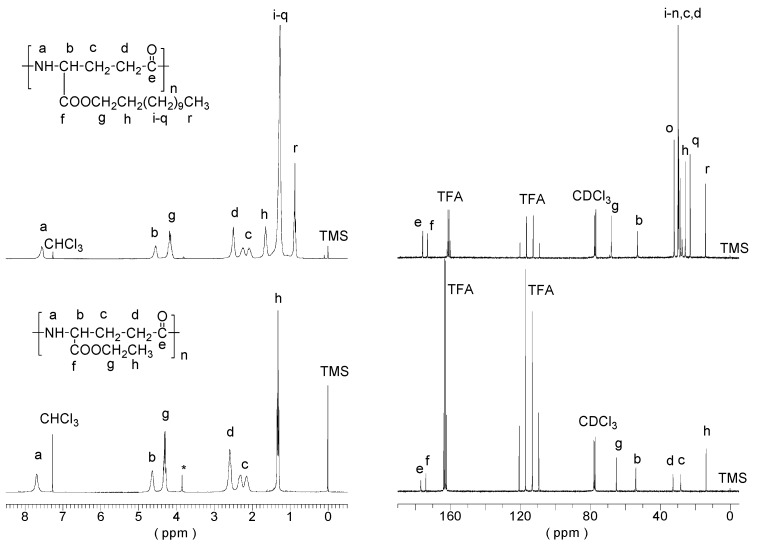
^1^H (Left) and ^13^C (Right) Nuclear Magnetic Resonance (NMR) spectra of Poly(α-dodecyl γ-glutamate) (PAAG-12) (top) and PAAG-2 (bottom) with peak assignments. CDCl_3_: deuterated chloroform; TFA: trifluoroacetic acid; TMS: tetramethylsilane.

Esterification of PGGA with ethyl bromide was checked by ^1^H NMR. Signals corresponding to methylene of ethyl group were compared with the α-CH group of PGGA to determine the degree of esterification. Both the ^1^H and ^13^C NMR spectra of PAAG-2 with peak assignments obtained at the end of the reaction are shown in Figure 1 (bottom). This polymer was then modified by transesterification with 1-dodecanol, where the ethyl group was totally replaced after 8 hours of reaction at 190 °C. The degree of transesterification was checked by comparing the signals of the α-CH group and the signals of the side methylenes and methyl of the dodecyl side chain. The ^1^H and ^13^C NMR spectra of purified PAAG-12 with peak assignments are shown in Figure 1 (top).

**Figure 2:**
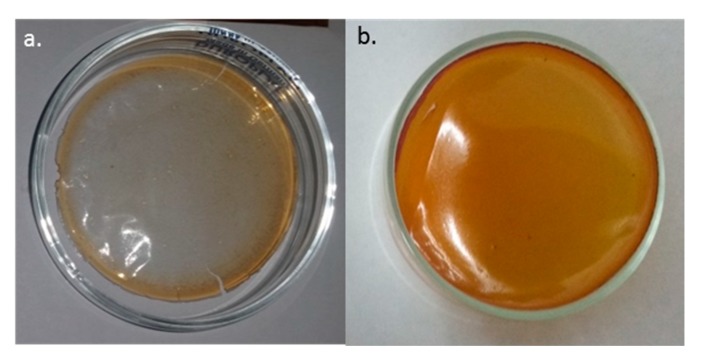
PAAG-12 control film (**a**) and film with 5% α-tocopherol (**b**).

Figure 2 depicts the prepared films. The main difference between them is the color. PAAG-12 films are transparent (Figure 2a), and when adding α-tocopherol, they have an amber tint (Figure 2b). They can be also told apart by touch, as control films are more brittle than the loaded ones, which present a gummous, moldable texture. 

The thickness of the PAAG-12 film with α-tocopherol was 0.142 mm. 

### 3.2. PAAG-12 Film Characterization Using TGA and DSC

**Figure 3:**
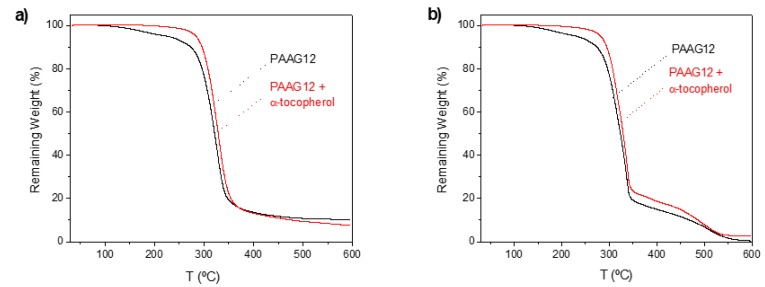
Thermogravimetric Analysis (TGA) of the PAAG-12 control film and the film charged with 5% α-tocopherol in (**a**) nitrogen and (**b**) air atmospheres.

Thermogravimetric analyses were performed in a nitrogen atmosphere for both PAAG-12 films and were compared, as depicted in Figure 3. Both nitrogen and air atmospheres were used to test the effect of different oxidant and inert atmospheres in the thermal degradation.

PAAG-12 shows a thermal decomposition that starts at about 200 °C, with a maximum decomposition rate at 226.4 and 229.1 °C for both nitrogen and air atmospheres (Table 1). It is observed that there is not much effect of the atmosphere in the thermal stability of this polymer. When these films were charged with α-tocopherol the thermal stability was substantially increased with an escalation of the onset temperature of about 58 and 50 °C, respectively, and slight rises in the maximum weight loss rates temperature. The enhanced thermal stability of this antioxidant on other polymers has been reported and correlated to its ability to efficiently deactivate all damaging free radicals, primarily alkylperoxyl and alkyl radicals generated in the thermal degradation processes [24]. These results are very promising because they show that the incorporation of a natural antioxidant such as α-tocopherol in this polymer improves its low thermal stability, thus allowing a better processability at higher temperatures.

In order to see if the thermal behavior of α-tocopherol or PGGA-12 was affected by the mutual blending, DSC thermograms were recorded. Unfortunately, no transitions were observed for both PGGA-12 and α-tocopherol or their blends, which prevents conclusions from being drawn from these DSC thermogram analyses.

### 3.3. Food Models

Two food models were used to test the antioxidant capacity of PAAG-12 films charged with 5% α-tocopherol. PLA films charged with the same amount of antioxidant were also used as a comparison.

#### 3.3.1. Food Simulant

The first food models were three types of solvents: water, 50% aqueous EtOH (as food simulants) and 95% aqueous EtOH (it was used to simulate extreme conditions such as very fatty foods, high temperatures, etc., and it is a concentration that can also be used). HPLC was used to determine α-tocopherol concentration.

Solvents in contact with PAAG-12 and PLA films were analyzed with HPLC and results demonstrated there was a progressive liberation of α-tocopherol in both ethanolic solvents, as summarized in Table 2. However, the migration was much higher for 95% EtOH than 50% EtOH. This was corroborated by ^1^H NMR analyses conducted to PAAG-12 films after application to food simulants (exhausted films); the remaining antioxidant in the polymer matrix is lower for 95% EtOH medium than 50% EtOH. This tendency was also observed in previous studies with synthetic phenolic antioxidants in PLA films [25]. Due to the lipophilic nature of α-tocopherol, no migration was observed when water was used as food simulant. Other substances added to PLA films such as cinnamaldehyde exhibited high rates of liberation at 20 days of contact [26] when using ethanol 50% as food simulant.

It was observed that PLA active films allowed higher migration of α-tocopherol to solvents, and this was especially marked with the 50% EtOH simulant. Several authors have found higher diffusion rates from films in higher amount of ethanol simulants, apparently due to the increased swelling of the polymer, thus creating void spaces favoring the migration of compounds [13,27].

When comparing both films, PAAG-12 exhibits lower release rates than PLA with the same simulant conditions. This indicates this novel biopolymer could be an alternative polymer matrix that can provide progressive release of antioxidants, for applications where slow liberations are intended.

#### 3.3.2. Primary Oxidation of O/W Emulsions Determined with Peroxide Value Assay

**Figure 4:**
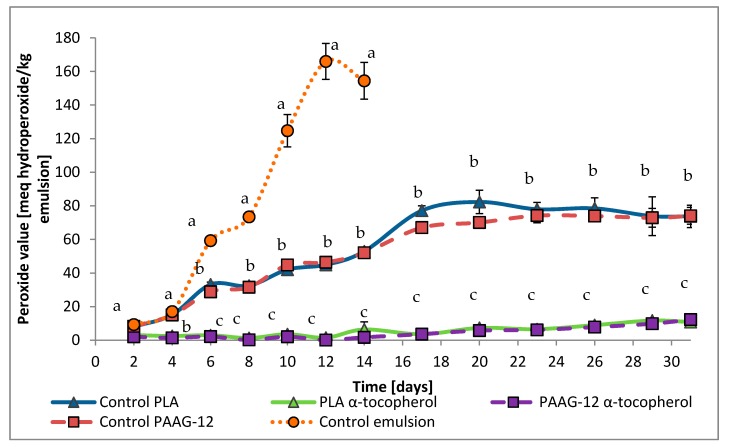
Primary oxidation value by peroxide assay of oil-in-water (O/W) emulsions protected by control emulsion, control PAAG-12, control PLA, PLA α-tocopherol and PAAG-12 α-tocopherol films. Samples with different letters denote significant differences (*p* < 0.01).

Thirty-one day tests were performed on emulsions protected with the films. Results were plotted and expressed as milliequivalents of hydroperoxide/kg emulsion with time, as can be seen in Figure 4.

In Figure 4, three differentiated behaviors are observed; on the one hand, the emulsion without any film (control emulsion) exhibits the highest oxidation rates. Oxidation measurements were stopped after 14 days as the peak of primary oxidation was reached. Further measurements would display parallel reactions of decomposition of chemical substances produced by oxidation, diminishing meq hydroperoxide, which would alter interpretation. 

On the other hand, there are the O/W emulsions protected with films that do not contain α-tocopherol. They undergo less oxidation than the control emulsion, implying that the polymer itself has an antioxidant effect. This represents a 72% reduction in oxidation at day 12 and 55.7% at the end of the experiment, day 31.

Finally, there are the films containing α-tocopherol. It should be noted that after 31 days they have only reached a peroxide value (PV) of 10 meq hydroperoxide/kg emulsion, which indicates that these active films are very powerful antioxidants, protecting the emulsion up to 92.6% compared to the control.

**Figure 5:**
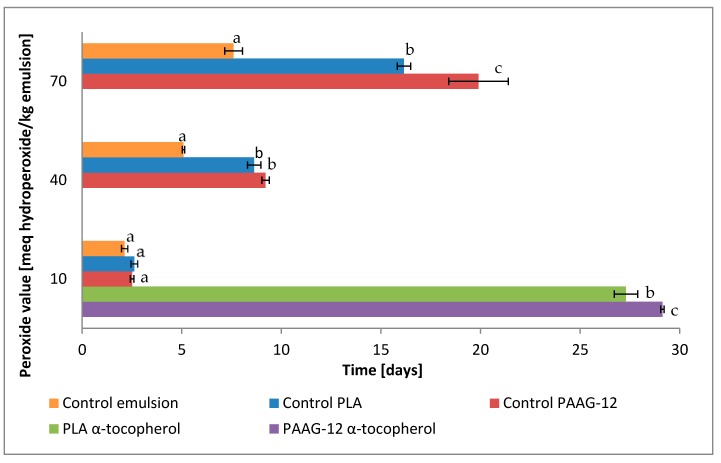
Time for samples to reach different peroxide values (graph). Samples with different letters denote significant differences (*p* < 0.01).

In order to provide a comparison of the films studied, the time to reach PV = 10, 40 and 70 meq hydroperoxide/kg emulsion has been calculated and is displayed in Figure 5. The different peroxide values were selected taking into consideration the compliance with the Codex Alimentarius, in which the maximum acceptable level of PV for refined vegetable oils is 10 milliequivalent/kg [28].

It can be observed that the addition of 5% of α-tocopherol to both films provides a strong protection, increasing the duration of the good condition of the emulsion (10 meq/kg) up to 29 days for PAAG-12 films and 27 days for PLA films. This is the reason for the lack of data for these two samples at 40 and 70 meq/kg in Figure 5, as those high oxidation values were not achieved.

The protection levels offered by the film without the antioxidant are only visible at higher peroxide values, as for the 10 meq/kg there were no significant differences between control emulsion and control films.

Previous studies conducted in our research team exhibited antioxidant protection with PLA active films in O/W emulsions up to 20 days [22] when using thyme and rosemary lyophilized extracts. 

Other authors conducted similar peroxide value experiments using marigold flower extract instead of α-tocopherol and prepared PLA pouches containing soybean oil [29] but surpassed the 10 meq/kg before day 5.

## 4. Conclusions

PAAG-12 was successfully synthesized from PGGA as demonstrated by NMR spectra. The increase of the onset temperature of PAAG-12 film when adding the natural antioxidant α-tocopherol validates the improvement of the thermal stability of the branched polymer, which entails a better processability for industrial applications for food packaging.

When assessing the first food model, higher migration of α-tocopherol from films to food simulants was observed when those simulants had higher ethanol concentration. This was demonstrated with HPLC analyses of food simulants and NMR of exhausted films. It was also revealed that PLA allowed a higher migration of antioxidant into the food simulant medium than PAAG-12 at short contact times, which demonstrates that this novel polymer is a promising matrix for active packaging applications.

The peroxide value assay of O/W emulsions proved the extraordinary protection levels of active films, capable of increasing the lifespan in compliance with the Codex Alimentarius (10 meq/kg) up to 29 days.

## Figures and Tables

**Table 1:** Values of TGA of the PAAG-12 control film, the film charged with 5% α-tocopherol and α-tocopherol film in nitrogen and air atmospheres.

Sample	Atmosphere	TGA ^a^		
		^o^T_d_ (°C)	^max^T_d_ (°C)	R_w_ (%)
PAAG-12	N_2_	226.4	325.6	10.2
	Air	229.1	337.4	0.4
PAAG-12 + 5% α-tocopherol	N_2_	284.1	328.9	7.5
	Air	279.4	338.7	2.6
α-tocopherol	N_2_	275.8	365.7	0
	Air	291.1	341.1	0.2

^a^ Thermal decomposition temperatures measured at 5% of weight loss (^o^T_d_ (°C)) and at maximum weight loss rate (^max^T_d_ (°C)). R_w_: weight (%) remaining after heating at 600 °C.

**Table 2:** Diffusion (*D*) and partition coefficients (*K_P,f_*) for α-tocopherol migration from PAAG-12 and PLA films into food simulants.

Polymer	Simulant	*K_P,f_*	D (m^2^/s)
PAAG-12	EtOH 50%	1.05	1.12 × 10^−11^
	EtOH 95%	0.37	5.58 × 10^−10^
PLA	EtOH 50%	0.94	1.11 × 10^−10^
	EtOH 95%	0.36	1.12 × 10^−10^
